# Cross-Linking of Polypropylene via the Diels–Alder Reaction

**DOI:** 10.3390/polym14061176

**Published:** 2022-03-15

**Authors:** Henky Muljana, Stefan Arends, Klaas Remerie, Gert Boven, Francesco Picchioni, Ranjita K. Bose

**Affiliations:** 1 Department of Chemical Engineering, Parahyangan Catholic University, Ciumbuleuit 94, Bandung 40141, Indonesia; 2 Department of Chemical Engineering, ENTEG, University of Groningen, Nijenborgh 4, 9747 AG Groningen, The Netherlands; stefanarends@gmail.com (S.A.); f.picchioni@rug.nl (F.P.); 3 SABIC, Plasticslaan 1, P.O. Box 117, 4600 AC Bergen op Zoom, The Netherlands; k.remerie@home.nl (K.R.); gert.boven@sabic.com (G.B.)

**Keywords:** polypropylene, cross-linked, Diels–Alder, furan-bismaleimide, thermoreversible

## Abstract

In this work, the possibility of preparing cross-linked polypropylene (PP) via Diels–Alder (DA) chemistry is explored. The overall strategy involves reaction of maleated polypropylene (the starting material), furfuryl amine (FFA), and bismaleimide (BM) as the cross-linking agent. The occurrence of reversible cross-linking was studied by checking the presence of relevant peaks in FTIR spectra, i.e., CH out-of-plane bending vibrations of the furan ring’s peak (γCH) at an absorption band of 730–734 cm^−1^, CH=CH of the BM aromatic ring’s stretching vibrations (υCH=CH) at an absorption band of 1510 cm^−1^, and the DA adduct (C-O-C, δDAring) at an absorption band of 1186 cm^−1^. In agreement with the spectroscopic characterization, the presence of a cross-linked network is also confirmed by rheology, namely the higher storage modulus (G′) compared with loss modulus (G″) value (G′ >> G″), as obtained via temperature sweep. Both the maleic anhydride (MA) content as well as the annealing temperature (50 °C and 120 °C) favor the DA reaction, while only partial de-cross-linking (retro DA) is observed at the higher temperature range of 150–200 °C. In addition, the products show higher mechanical robustness and thermal stability compared to the starting material.

## 1. Introduction

Since the discovery of the Ziegler–Natta catalyst in 1954, polypropylene has been commercially produced on a large scale worldwide and its production volume is increasing each year. Currently, PP has become one of the most essential thermoplastics in daily application [1,2]. This is not surprising, since besides its favorable price, PP has superior properties such as high toughness, good impact/rigidity profile, high tensile strength, good flexibility, low density, and good chemical and heat resistance compared to other thermoplastic materials [1,2,3,4,5]. Therefore, PP has found a wide range of applications in automotives, textiles, household products, medical applications, packaging, and adhesives (the latter especially for atactic PP) [1,3,6,7].

Despite its superior properties, chemical modification of PP is still often required in order to improve some drawbacks in its properties and to expand its applicability in other areas. Among others, the cross-linking of PP has gained significant interest from many researchers worldwide. The technology offers the possibility to improve temperature stability, to increase electrical discharge and chemical resistance, to achieve high melt strength, and to increase mechanical resistance towards creep and stress cracking [8,9,10]. The conventional process to cross-link PP involves a macroradical formation through various possible routes, for instance via thermal decomposition of peroxides, high energy irradiation (gamma and electron beams), ultraviolet (UV) radiation with UV sensitizer, and silane grafting with moisture cross-linking [8,9,11,12]. However, current processes suffer from the degradation of the PP backbone, and this further hinders the commercial application of this technology on the industrial scale [8,9]. Therefore, there is a strong incentive to find another possible route to produce a cross-linked PP with less degradation in the PP backbone.

The Diels–Alder (DA) reaction has been widely used as a cross-linking tool in many polymer systems because of its fast kinetics and relatively mild reaction conditions. Among others, the DA reaction using a furan and a bismaleimide as a cross-linking agent in various polymers has been extensively explored in the last decade. A few successful examples are the cross-linking of polyketone functionalized with furfurylamine and bismaleimide [13,14,15], the cross-linking of polymethacrylate containing furan and maleimide functionalities with a long-chain lauryl methacrylate incorporated in the polymer network [16], the cross-linking of polyurethanes synthesized from a trifunctionalized isophorone diisocyanate (IPDI) and polypropyleneglycol prepolymer with furfuryl alcohol and maleimide [17], and the cross-linking of maleated ethylene propylene rubber (EPM) functionalized with furfurylamine and bismaleimide [18,19]. Another example of furan maleimide DA cross-linking in various polymeric systems (such as furyl telechelic polyesters with di/trifunctional maleimides, furan-modified poly(ε-caprolactone)-urethane and BM, etc.) and an in-depth explanation on furan maleimide containing polymeric materials in the biomedical applications are clearly described in the elegant review by Gandini [20] and Gevrek [21], respectively. Trends on the Diels–Alder in polymer chemistry and its potential future application have also been clearly described in the recent publication [22].

Despite the successful application of DA cross-linking using furan and bismaleimide in many polymer systems, to the best of our knowledge not many studies have been carried out in polyolefins. Until now, only the work of Magana et al. reported the cross-linking of Lotader (polyethylene-co-glycidyl methacrylate), in which furan and maleimide were grafted on the Lotader via DA reaction between 3-(2-furyl) propanoic acid and the synthesized 1,1-maleimido-undecanoic acid dienophile [23]. The result of their work indicates that the DA chemistry with furan and BM as the diene and dienophile pair can also be applied as a new synthetic route to produce a cross-linked PP. The product is expected to have improved properties compared with those synthesized via conventional cross-linking methods (see above).

**Scheme 1:**
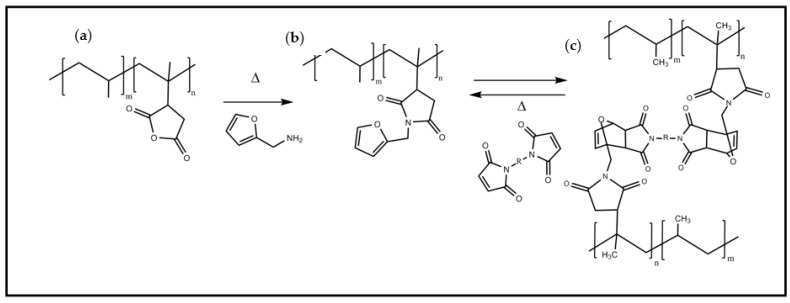
The functionalization of PPgMA (**a**) with furfurylamine (FFA) (**b**) followed with Diels–Alder reaction with bismaleimide (BM) (**c**).

The aim of this work is to investigate the application of DA chemistry on the cross-linking of PP synthesis using FFA and BM. The proposed reaction pathway is shown in Scheme 1 where a similar approach has been reported and successfully applied in the DA cross-linking of maleated EPM rubber [18]. Initially, PP has to be functionalized with maleic anhydride to form maleated PP (Scheme 1a). This step has been intensively investigated and numerous reports are available in open literature [24,25,26,27,28,29,30]. Thus, the synthesis of polypropylene graft maleic anhydride (PpgMA) is outside the scope of this work and we decided to use commercially available PpgMA as the starting material. As a consequence, this work focuses on the modification of maleated PP via two consecutive steps, which are the grafting of furfuryl amine (FFA) into anhydride rings of PpgMA (Scheme 1b) to form succinic imide; and the DA reaction between the grafted furan (an electron-rich diene) with bismaleimide (BM) (an electron-poor dienophile, Scheme 1c).

In this work, the influence of different maleic anhydride (MA) content in the starting material and the temperature dependence of DA chemistry on the extent of cross-linking were evaluated using Fourier transform infrared (FTIR) spectroscopy, oscillatory shear rheology, solubility measurements, and differential scanning calorimetry (DSC). Furthermore, the thermal stability of the cross-linked products was analyzed using thermal gravimetry analysis (TGA).

## 2. Materials and Methods

### 2.1. Materials

Polypropylene-grafted maleic anhydride (PPgMA, average M_n_ 3900 g/mol, average M_w_ 9100 g/mol, and 8–10 wt. % maleic anhydride) was purchased from Sigma Aldrich (Munich, Germany).

Polypropylene-grafted maleic anhydride with higher molecular weight produced by Exxon Mobil USA (PPgMASB, M_n_ 50–100 kg/mol, and 0.5–1 wt. % maleic anhydride) was kindly supplied by SABIC (Geleen, The Netherlands). Analytical-grade furfurylamine (FFA, >99%) was purchased from Sigma Aldrich (Munich, Germany) and was freshly distilled before the experiment. Analytical-grade 1,1′-(methylenedi-4,1-phenylene) bismaleimide (BM, 95%), tetrahydrofuran (THF, >99.9%), and chloroform (CHCl_3_, >99%) were purchased from Sigma Aldrich (Munich, Germany). 1–2 dichlorobenzene (DCB, >99%) was purchased from Fluka (Landsmeer, The Netherlands). Antioxidant AOB225 (CAS Number 9421-57-8) was kindly supplied by SABIC (Geleen, The Netherlands).

### 2.2. Experimental Procedures

#### 2.2.1. Functionalization of Maleated PP (PPgMA/PPgMASB) with FFA

PPgMA/PPgMASB (15 g), and 4 equivalents of FFA based on MA content in PPgMA/PPgMASB were mixed in a Brabender kneader for 10 min with rotational speed of 50 rpm and temperature of 160 °C. The solid product was grinded and washed using boiling THF at 170 °C in a SOXTEC apparatus for 3 h to remove the excess of the reagent. The samples were then dried in a vacuum oven at 50 °C until no further weight loss was observed. Next, the dried products were compressed at 175 °C and 100 bar for 30 min to complete the reaction by closing the amine aromatic ring [18]. Slightly brown products were obtained after the product workup.

#### 2.2.2. Bismaleimide Cross-Linking with Functionalized PPgMA/PPgMASB

The functionalized PPgMA product (2 g), 0.5 equivalent of BM based on the theoretical amount of furan in the functionalized product, antioxidants (AOB225, 4 mg) and CHCl_3_ (20 g) as the solvent were added into a 25 mL round-bottom flask. Afterwards, CHCl_3_ (20 g) was added into the flask. The mixture was heated at 50 °C in a water bath and stirred for 3 h. After the reaction, CHCl_3_ was separated from the product by slow evaporation in the fume cabinet for 24 h. The solid product was annealed at different temperatures for 24 h.

#### 2.2.3. Solubility Test of the Cross-Linked Products

Solubility tests were carried out to qualitatively determine the extent of cross-linking or de-cross-linking of the final product after annealing step at different temperatures. The annealed products (0.05 mg) and dichlorobenzene (DCB, 2 g) as the solvent were added into a small testing bottle (5 mL) and were heated up in an oven at 120 °C for 24 h. The photograph was taken prior to heating up in the oven (t = 0) and after heating up for 24 h (t = 24 h) to check on solubility of the product.

#### 2.2.4. Analytical Equipment

FTIR spectra were acquired on a Shimadzu IRTracer-100 (Kyoto, Japan) equipped with an attenuated total reflectance (ATR) Golden Gate Specac Golden Gate ATR Top and West 6100+ temperature controller (Philadelphia, PA, USA) with a diamond crystal. FTIR measurements were taken with 64 scans in the absorption range of 4000 to 500 cm^−1^ with a resolution of 4 cm^−1^. The spectra were deconvoluted (R > 0.95) to calculate the change in the intensity of the relevant peaks.

TGA analysis was conducted on a PerkinElmer TGA 4000 (Waltham, MA, USA). The samples (10 mg) were heated to 700 °C in an inert atmosphere with a heating rate of 10 °C min^−1^. DSC was performed on a PerkinElmer DSC 7 Pyris 1 (Shelton, ST, USA) under a nitrogen atmosphere. The sample (10 mg) was inserted inside a sealed aluminum pan and heated from 25 to 250 °C with a heating rate of of 5 °C/min. Next, the sample pan was cooled down to 25 °C with a cooling rate of 5 °C/min. The heating and cooling step were repeated two times for each sample.

Elemental analysis for C, H, N was performed using a Euro EA 3000 Eurovector S.P.A Elemental Analyzer (Langenselbold, Germany).

The rheology of the materials was measured using Haake Mars III (Thermo Fisher Scientific, Karlsruhe, Germany) equipped with Controlled Test Chamber (CTC). The samples (diameter of 2.5 cm and thickness of 1 mm) were prepared with compression molding using a Taunus Ton press type VS up 150 A at temperature of 155 °C and pressure of 100 bar for 10 min, and subsequently the press was cooled to room temperature using cooling water for approximately 20 min. The shear rheology was measured using 1% of strain, which is in the linear viscoelastic regime for all samples, and duplicate measurements were taken to ensure the reproducibility of the shear rheology test. Temperature-sweep experiment was performed in temperature range of 150–200 °C (5 °C/min) and angular frequency (ω) of 1 rad/s. Frequency-sweep experiments were performed in the frequency range of 0.01–100 rad/s and temperature of 160 °C. The storage modulus (G′), loss modulus (G″), loss tan (δ), complex modulus (|G*|), and the complex viscosity (|η*|) were measured as functions of temperature and ω.

## 3. Results

A series of experiments was performed based on the proposed reaction scheme (Scheme 1). The influence of different MA intakes on each reaction step, i.e., grafting of FFA and DA cross-linking with BM, was evaluated at low (0.5–1 wt. %, PPgMASB) and high MA content (8–10 wt. %, PPgMA). In addition, the effect of annealing temperature on the DA cross-linking was also studied for both PPgMA and PPgMASB products.

Details of the experimental conditions for each step and the corresponding product code are given in Table 1. The experimental results for each reaction step are discussed in the following section.

### 3.1. Grafting of Furfurylamine (FFA) onto PPgMA and PPgMASB

**Figure 1:**
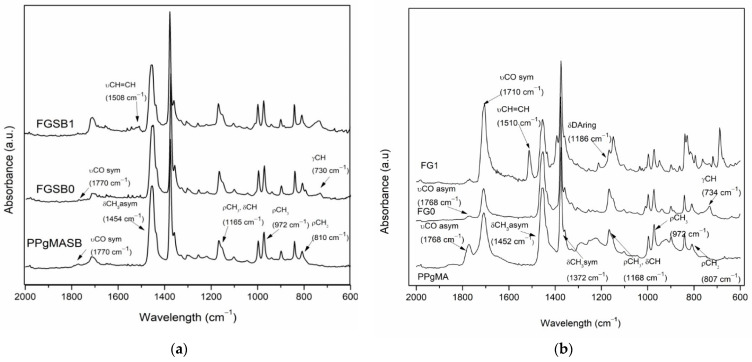
FTIR spectra of PPgMASB and its derivatives (**a**); PPgMA and its derivatives (**b**).

The insertion of the furan group into anhydride rings and resulting succinimide was verified with FTIR analysis. FTIR spectra of maleated PP are shown in Figure 1. Both PPgMASB (Figure 1a) and PPgMA (Figure 1b) spectra show several peaks, which are typical for PP and the grafted maleic anhydride groups. PP spectra have strong absorption bands at 1450–1453 cm^−1^ (CH_3_ asymmetric bending, δCH_3_ asym), 1370–1375 cm^−1^ (CH_3_ symmetric bending, δCH_3_ sym), 1165 cm^−1^ (CH_3_ rocking, ρCH_3_ and CH bending, δCH), 968–970 cm^−1^ (CH_3_ rocking ρCH_3_) and 806–810 cm^−1^ (CH_2_ rocking, ρCH_2_), as shown in Figure 1 [26,31]. The presence of the anhydride group is clearly shown from the peaks at the absorption band of 1768–1770 cm^−1^ (C=O asymmetric stretching, υCOasym) and 1710–1715 cm^−1^ (C=O symmetric stretching, υCOsym) (Figure 1) [18,24,25,26,27].

After grafting with FFA, the changes of several peaks are clearly visible in the spectra of FGSB0 (Figure 1a) and FG0 (Figure 1b). The strong absorption band at 1768–1770 cm^−1^ (C=O asymmetric stretching of the succinic anhydride, υCOasym) (Figure 1a) decreases when the furan group is grafted (Figure 1a). Moreover, an additional CH out-of-plane bending vibration of the furan ring peak appears at the absorption band of 730–734 cm^−1^ (γCH, Figure 1a) [32,33]. Similar changes also appear at the same peak’s absorbance in the spectra of furan-grafted PPgMA (FG0, Figure 1b), albeit with larger intensity compared with those of PPgMASB (FGSB0, Figure 1b). The difference in the intensity may be related to the different MA content in the starting material and may eventually influence the grafting efficiency. It is reasonable to assume that more furan can be grafted at a higher MA content, where a similar trend is shown in the DA cross-linking of maleated EPM rubber [18].

The grafting efficiency was calculated based on the C, H, N composition of the starting material and the grafted products, as obtained from the elemental analysis, and the results are given in Table 2.

Elemental analysis of the starting material (Table 2) shows that PPgMA has higher MA content (8.9 wt. %) compared with PPgMASB (0.58 wt. %). The calculated MA content is in agreement with that provided by the manufacturers. By assuming that the amount of furan grafted on PPgMA or PPgMASB is equal (in terms of mol %) with the N content in the products (Scheme 1), it is obvious that more furan is grafted in PPgMA (FG0, N% = 0.34%) compared with PPgMASB (FGSB0, N% = 0.08%). This is in line with the findings in the FTIR spectra (see above). Although more furan can be grafted, it is clear that the grafting of furan in PPgMA is still far from quantitative. Only 26.3% of furan (based on N content) was grafted to the PPgMA (FG0), while for PPgMASB, the conversion is significantly higher (96.2%).

Significantly higher grafting conversion compared with PPgMA is also reported by Polgar et al., in which about 93% of furan can be grafted in the maleated EPM rubber (MA content = 2.1 wt. %) [18]. In their experiments, the grafting reaction was conducted in THF solution and a reaction time of five hours, while in our case, the grafting reaction is conducted in the melt with a relatively short time (10 min). Although the proper comparison is cumbersome (solution versus melt reaction), this implies that in our case, mass-transfer limitation may take place and further hinder the overall reaction rate. Since the optimization of the grafting conversion was not the aim of this work, we proceeded to investigate the DA cross-linking of the prepared polymers. These results are shown and discussed in the following section.

### 3.2. Cross-Linking via Diels–Alder of Furan-Bismaleimide

After the furan-grafting reaction, the intermediate products (FGSB0 and FG0) were further reacted with BM using DA chemistry. The presence of BM in the products was verified by the appearance of the peak at an absorption band of 1510 cm^−1^, which corresponds to CH = CH of the BM aromatic ring’s stretching vibrations (υCH=CH) as observed in Figure 1a for FGSB1 and Figure 1b for FG1 [34]. The DA adduct (C-O-C, Scheme 1c), however, is only present in FG1, as shown from the shoulder around the absorption band of 1186 cm^−1^ (δDAring, Figure 1a) [13,18,35]. The absence of this shoulder in the FGSB1 spectrum indicates that the Diels–Alder reaction between furan and BM is probably not yet happening. This may be due to the low amount of furan grafted to the FGSB0 which eventually reduces the reactivity in the DA reaction.

**Figure 2:**
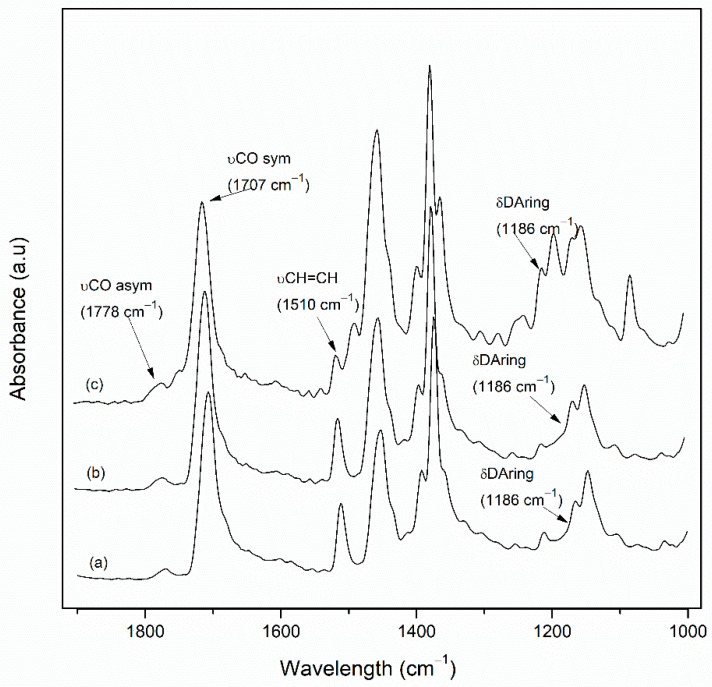
FTIR spectra of FG1 (**a**), FG1A50 (**b**), and FG1A120 (**c**) in the absorption band of 1000–1900 cm^−1^.

**Figure 3:**
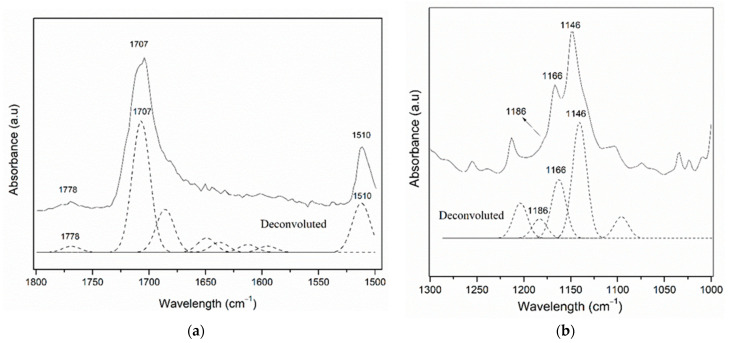
An example of a deconvoluted spectrum of FG1 within the wavelength of 1500–1800 cm^−1^ (**a**); and 1000–1300 cm^−1^ (**b**).

Moreover, changes in the intensity of the two peaks (1510 cm^−1^ and 1186 cm^−1^) associated with the cycloadduct formation at different annealing temperatures (50 °C and 120 °C) were observed (Figure 2). Figure 2 shows the FTIR spectra of FG1 and the annealed products (FG1A50 and FG1A120) in the absorption range of 1000–1900 cm^−1^. It seems that the intensity of the peak at 1510 cm^−1^ decreases with temperature, while the intensity of the peak at 1186 cm^−1^ increases at a higher temperature. The quantification of these changes was made based on the deconvolution of FTIR spectra for all products (an example is provided in Figure 3). The intensity of each peak was normalized to the intensity of the absorption peak at 1707–1710 cm^−1^. This peak is ascribed to C=O symmetric stretching (υCO sym) of anhydride and BM rings [36,37] and chosen as the standard since its intensity remains constant, as the peak does not significantly change for unreacted and reacted BM and the relatively high melting point prevents any significant loss of BM during mixing. Table 3 shows the normalized intensity ratio of the two peaks at absorption bands of 1510 cm^−1^ (I_1510_/I_1707_) and 1184 cm^−1^ (I_1184_/I_1707_) for FG1, FGSB1, and their annealed products.

As shown in Table 3, it is clear that the intensity of the peak at 1510 cm^−1^ for FG1 decreases with the annealing temperature from 0.377 (FG1) to 0.255 (FG1A120), suggesting the decrease in the intensity of CH=CH BM aromatic rings due to cycloadduct formation (Scheme 1c). Furthermore, the DA adduct between furan and bismaleimide is already present in FG1 before annealing (Table 3) and the intensity of the peak (I_1184_/I_1707_) increases at a higher annealing temperature from 0.077 (FG1) to 0.618 (FG1A120). The increase in the intensity may be related with the higher degree of cross-linking formed at higher annealing temperature.

In contrast with FG1, there is no significant difference of I_1510_/I_1707_ for FGSB1 and its annealed products (Table 3). However, small changes are observed in the DA adduct (I_1184_/I_1707_) at the highest annealing temperature (T = 120 °C, FGSB1A120), indicating the presence of DA cross-linking, albeit lower in extent compared with FG1A120. These observations (Table 3) clearly indicate the positive influence of temperature on the DA reaction within the experimental window.

**Figure 4:**
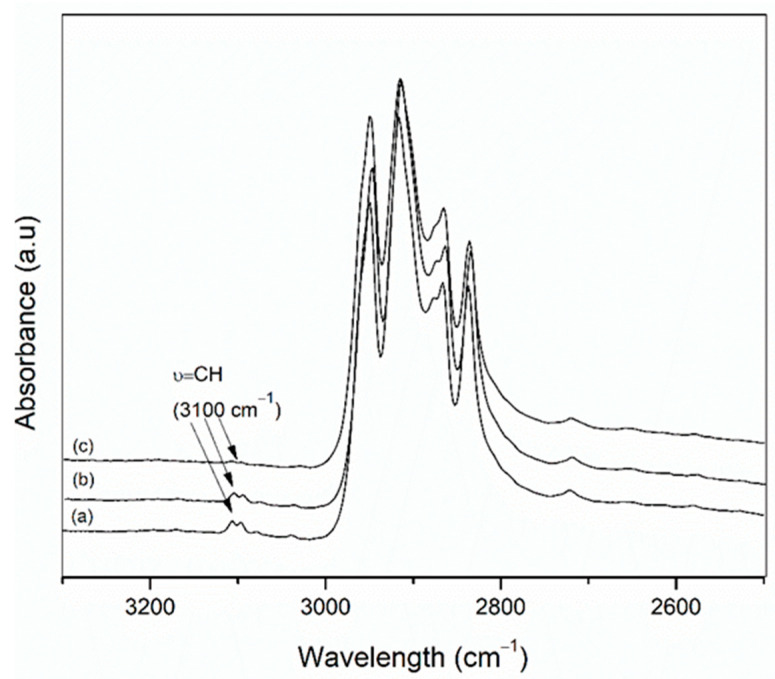
FTIR spectra of FG1 (**a**), FG1A50 (**b**), and FG1A120 (**c**) in the absorption band of 2500–3300 cm^−1^.

In addition, the changes in the intensity were also present in the peak at an absorption band of 3100 cm^−1^ (Figure 4). This peak, ascribed to =CH stretching vibrations of BM rings (υ = CH) [38], decreases at a higher annealing temperature as in agreement with the changes of the peak at 1510 cm^−1^.

**Figure 5:**
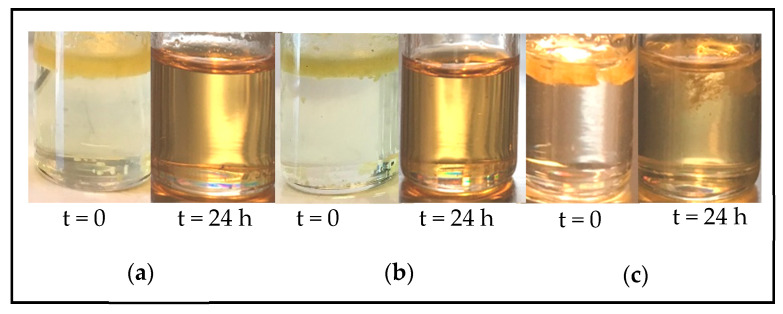
Solubility of FG1 (**a**), FG1A50 (**b**), and FG1A120 (**c**) in dichlorobenzene at 120 °C for 24 h.

More evidence to show an increase in the degree of cross-linking is obtained from the solubility test of the annealed products. Figure 5 shows the solubility of FG1 and its annealed products at room temperature (t = 0) and after 24 h at 120 °C (t = 24 h). It is clear that FG1 and FG1A50 samples are still soluble in DCB, while the FG1A120 sample is partially soluble in DCB. The difference in solubility is strongly related with the presence of cross-linking in the sample. It is then evident that the FG1A120 sample has a higher degree of cross-linking compared with the other products (FG1 and FG1A50).

**Figure 6:**
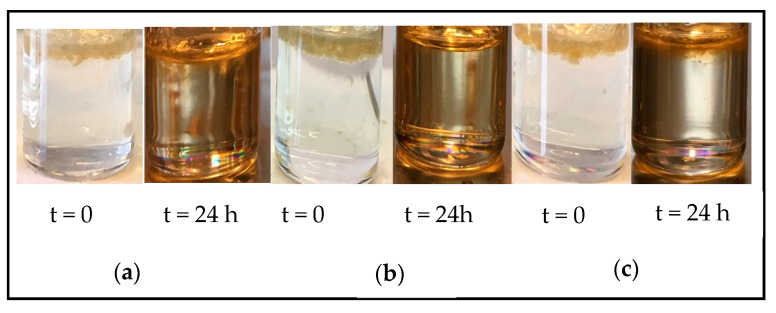
Solubility of FGSB1 (**a**), FGSB1A50 (**b**), and FGSB1A120 (**c**) in dichlorobenzene at 120 °C for 24 h.

The solubility test was also performed on the FGSB1 and its annealed products (Figure 6). As expected, both FGSB1 and FGSB1A50 are soluble in DCB. It is clear that FGSB1A120 has less solubility in DCB compared with the other two samples (FGSB1 and FGSB1A50). This suggests the formation of a cross-linked network in the FGSB1 after annealing. This is in agreement with the FTIR results.

#### Rheology and Thermal Stability of the Cross-Linked Products

**Figure 7:**
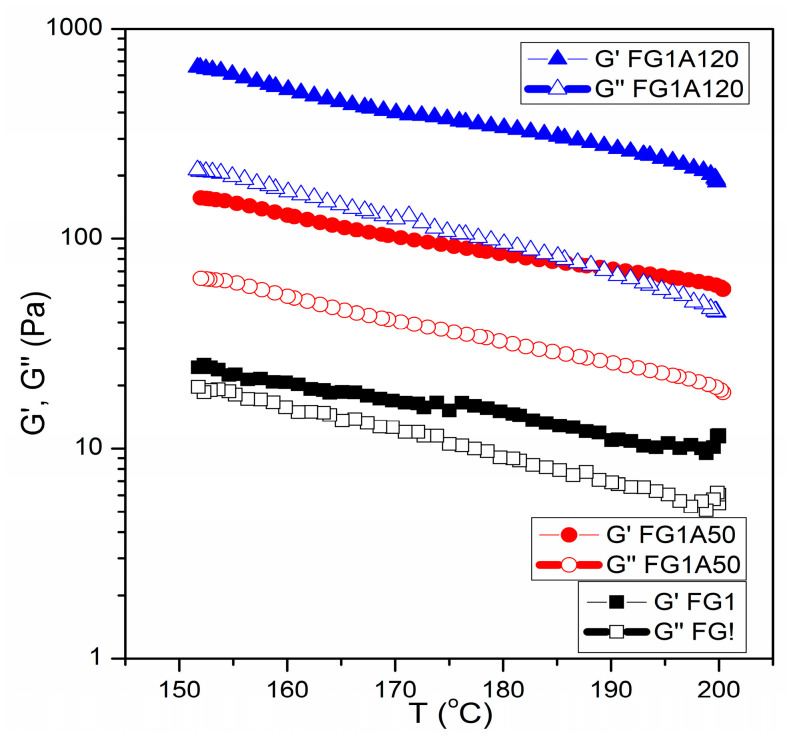
Storage (G′) and loss (G″) modulus of FG1, FG1A50, and FG1A120 at various temperatures, and angular frequency (ω) of 1 rad/s.

Thus far, all the findings from both FTIR and the solubility test show evidence of DA cross-linking between furan and bismaleimide in the PPgMA backbone and the positive influence of the annealing temperature towards the degree of cross-linking. However, we also notice that DA cross-linking still increases with temperature even at 120 °C, which is in apparent contrast with the reversibility behavior (in terms of temperature range) of other systems. It is evident that the equilibrium in DA reaction between furan and bismaleimide is reversed towards de-cross-linking already at a temperature of 100 °C and higher [16,20,23]. Therefore, temperature-sweep oscillatory rheology measurements were performed on FG1 to investigate the influence of the higher temperature (T > 120 °C) on the cross-linking. The result is shown in Figure 7.

As can be seen in Figure 7, the storage modulus (G′) is always higher than the loss modulus (G″) (G′ >> G″) and a decrease in G′ with temperature (T = 150–200 °C) is clearly visible in FG1 (Figure 7, black line). These observations imply that the FG1 product is still highly cross-linked (G′ >> G″) even at the highest temperature in the range (T = 200 °C) [16]. A decrease in G′ suggests a lower degree of cross-linking values compared with that at the lower temperature (T < 200 °C). The latter indicates that at temperatures higher than 150 °C, only partial de-cross-linking takes place via retro DA reaction. It is possible that within the measurement window, the partial de-cross-linking may happen due to the aromatization process of the DA adduct at a high temperature (T > 150 °C) and may eventually lead to more thermostable end products [20].

Furthermore, we performed thermal analysis using DSC to evaluate the reversibility of the cross-linked products (see Figure S1 of the Supporting Information). The thermogram shows two distinguished peaks, which are the melting (T_m_ = 150–152 °C) and the crystallization peaks (T_c_ = 119–120 °C) of the PP backbone, while no exothermic and endothermic transition of DA and retro DA appears, respectively. The absence of DA and retro DA transition indicates that reversibility of the cross-linked products cannot be observed from the DSC measurement. This finding is in contrast to previous work by our research group involving the same reaction step using maleated EPM rubber, where full de-cross-linking is observed already at 150 °C. The difference in the results may be related to a different polymer structure of both materials, i.e., semicrystalline (FG1) versus amorphous (maleated EPM rubber) [18]. In the semicrystalline polymer, the cross-linking and de-cross-linking of the product is not solely determined by the DA and retro DA reaction but also influenced by the chain mobility of the polymer. The latter indicates that both the amorphous (glass transition) [39] and crystalline part of the polymer (melting, and crystallization) [40,41] have an obvious influence on chain (segmental) mobility, and this might in turn affect the strain on the cross-linking points—in this case, the DA adduct.

In addition, a similar trend as for FG1 is also detected in both annealed products (FG1A50, red line; and FG1A120, blue line, see Figure 7). Both FG1A50 and FG1A120 show higher G′ compared with G″ (G′ >> G″) and no crossover between the two values detected in the measurement range. It is important to note that both G′ and G″ values of the annealed product are significantly higher compared with the ones of FG1. This may be related to the increase in the degree of cross-linking after annealing and eventually affects the viscoelastic properties of the products.

**Figure 8:**
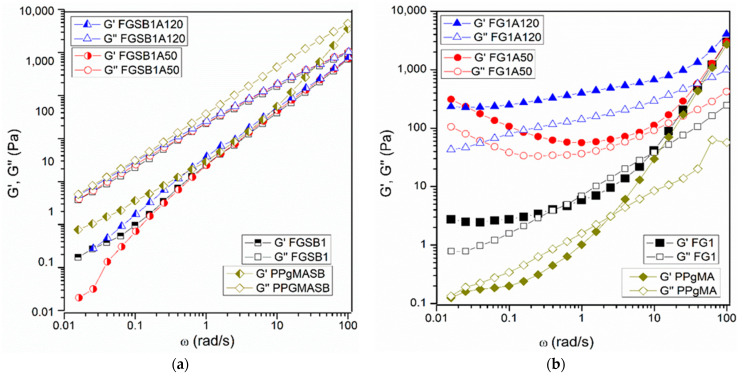
Storage (G′) and loss (G″) modulus of PPgMASB and its derivatives (FGSB1, FGSB1A50, FGSB1A120) (**a**); PPgMA and its derivatives (FG1, FG1A50, FG1A120) (**b**) at various angular frequencies (ω, rad/s) measured at 160 °C.

Further changes in the melt rheological properties (i.e.,: elasticity, melt strength, complex viscosity, and stiffness) of the starting materials and the modified products were further explored with frequency-sweep measurements. Figure 8 shows the comparison of storage (G′) and loss (G″) modulus for FG1, FGSB1, and their annealed products at different frequencies (0.01–100 rad/s). The measurement was taken at 160 °C. The rheograms of FGSB1 and the annealed products (Figure 8a) show an increase in both G′ and G″ with the frequency. The same trend is also observed in the starting material (PPgMASB, Figure 8a). Moreover, a typical liquid-like melt behavior (G″ >> G′) is shown in PPgMASB, FGSB1, and its annealed products (Figure 8a) [42].

In contrast with previous observations, the higher G′ values compared with G″ values are observed in FG1 and its annealed products, especially at the low-frequency region (Figure 8b). When comparing with the rheograms of the starting material (PPgMA, Figure 8b), it is obvious that after modification, the rheological properties at the low-frequency region are shifted from liquid-like (G″ >> G′) into solid-like melt behavior (G′ >> G″) [42]. This change is undoubtedly due to the presence of the cross-linked network in the product. Furthermore, the cross-over point (G′=G″), which is spotted in the starting material (PPgMA), gradually disappears from the rheogram, especially in the annealed product (Figure 8b). This, again, may be attributed to the higher degree of cross-linking present in the polymeric network of the annealed products and the aromatization of the DA adduct at a higher temperature.

**Figure 9:**
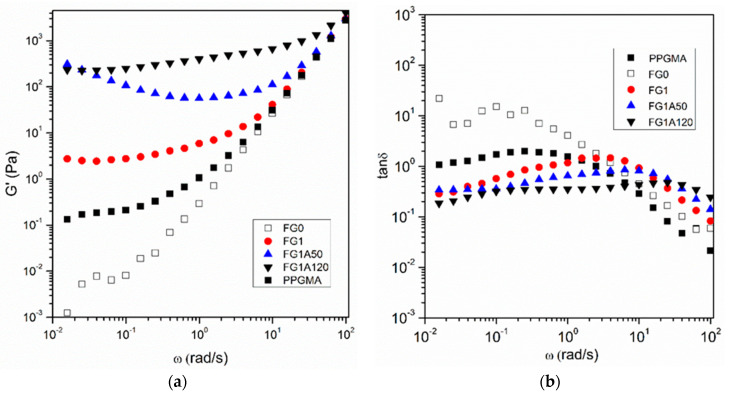
Storage (G′) modulus (**a**) and loss tangent (tan δ) (**b**) of PPgMA and its derivatives at various angular frequency (ω, rad/s) and measured at temperature of 160 °C.

The melt elasticity changes due to the chemical modification of the starting material (PPgMA) are presented in Figure 9. It is apparent that the G′ value of FG0 is somewhat lower compared to PPgMA. This may be related to the occurrence of β-scission in the PP backbone during the grafting reaction at a high temperature (T = 160 °C) [10] and may reduce the melt elasticity in the grafted product (FG0). The lower melt elasticity is also observed in PPgMASB after the furan-grafting reaction (FGSB0), as indicated by the lower G′ value of FGSB0 compared with the G′ value of PPgMASB (Figure S2 in Supporting Information). Despite the loss in melt elasticity, a remarkable increase in the G′ values is observed in the FG1 and its annealed products, especially at the low-frequency region. The maximum elasticity was achieved by FG1A120. An increase in melt elasticity is strongly related with the change in the chain mobility after the cross-linking reaction [43,44,45].

In addition, an increase in melt elasticity may lead to higher melt strength, as indicated by the decrease in loss tangent (tan δ, Figure 9b) at the low-frequency region [42]. As expected, all the cross-linked products (FG1, FG1A50, and FG1A120) have a lower tan δ compared with PPgMA and FG0. This suggests that the presence of the cross-linking network has successfully improved the melt strength of the end products. In this case, FG1A120 has the highest melt strength.

**Figure 10:**
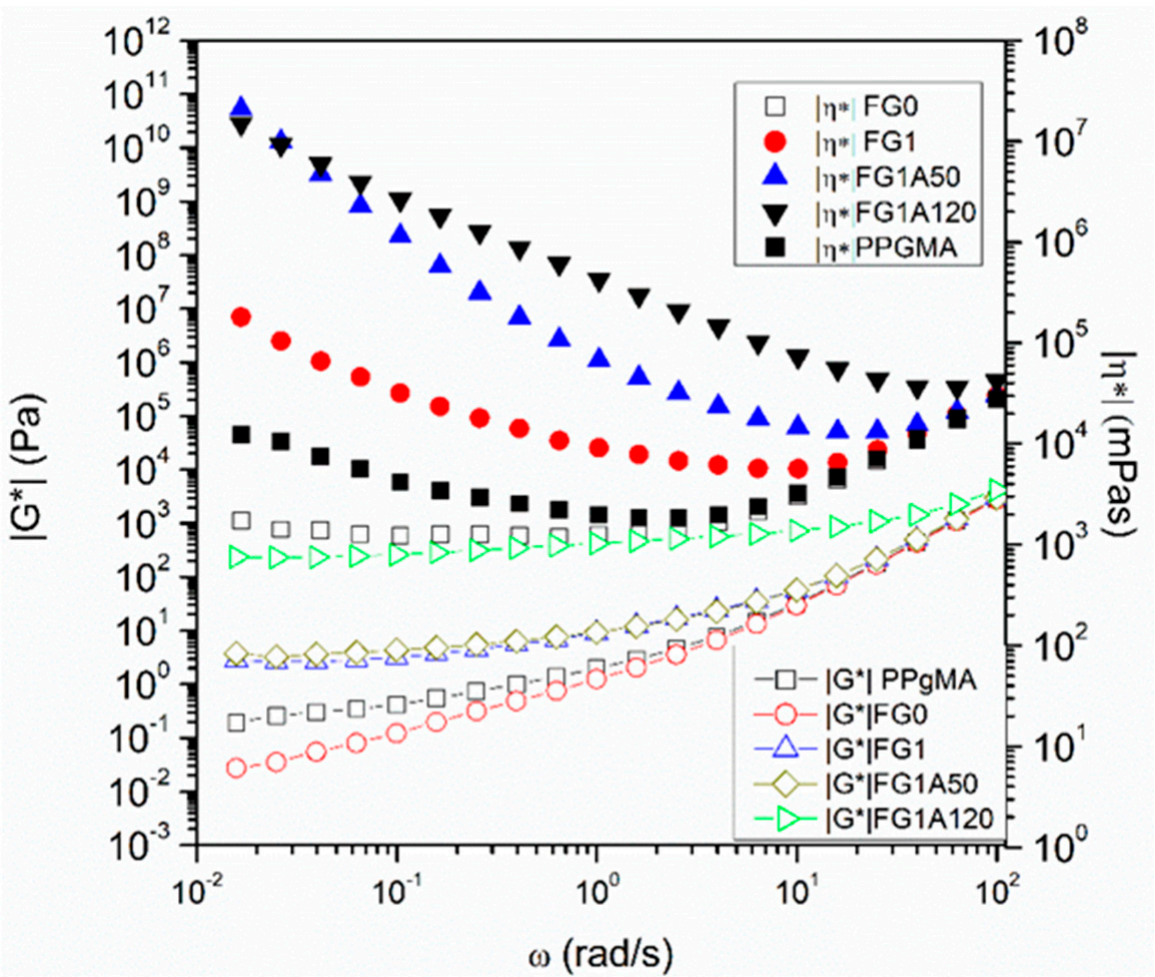
Complex modulus (|G*|) and complex viscosity (|η*|) of PPgMA and its derivatives at various angular frequencies (ω, rad/s) and measured at temperature of 160 °C.

In addition to the melt strength, the rigidity of the products is also significantly improved after cross-linking and annealing at a higher temperature (Figure 10). This is demonstrated with the higher complex modulus (|G*|) [16] values of the modified products compared with PPgMA and FG0 at the low-frequency region. These results signify the benefits of using DA cross-linking technology on the mechanical robustness of the final products.

The effect of DA cross-linking on the complex viscosity (|η*|) of all the products is shown in Figure 10. In agreement with the other parameters, the |η*| value is expected to be higher due to the formation of a cross-linked network after DA reaction, as shown in Figure 10 for FG1 and its annealed products [10]. Surprisingly, although no DA cross-linking is detected in the FGSB1 and FGSB1A50 as measured with FTIR, a higher |η*| value of the two products compared with PPgMASB and FGSB0 is observed (see Figure S3, Supporting Information). We assume that this may be related with the decrease in the chain mobility in the polymeric network after modification, resulting in an increase of the |η*| value [45]. When the cross-linked network is formed, the chain mobility is restricted and eventually leads to even higher |η*| values (Figure S3).

**Figure 11:**
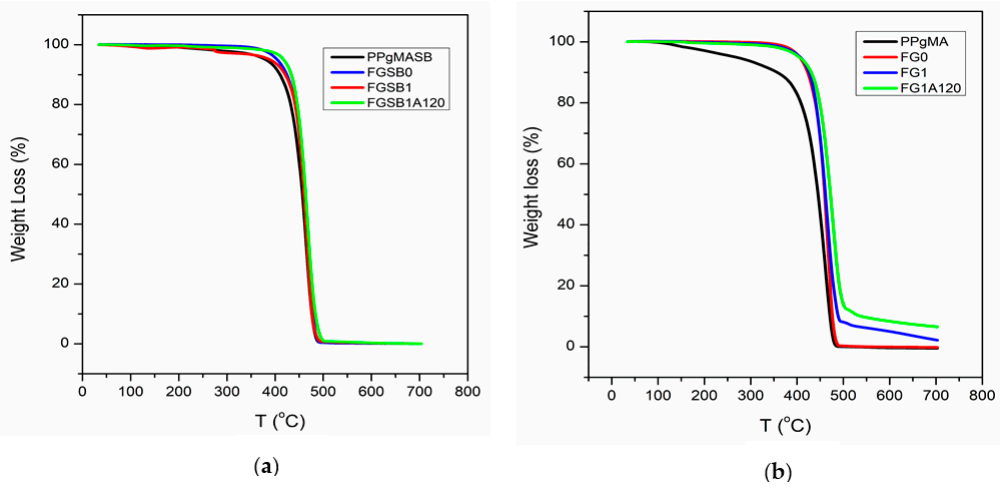
TGA thermograms of PPgMASB (**a**) and PPgMA (**b**) and their modified products.

In addition to the changes in the rheological properties, the formation of the cross-linked network in the PP structure may also affect the thermal stability of the product. The TGA thermograms of the PPgMASB and PPgMA with their modified products are shown in Figure 11a,b, respectively. PPgMASB (Figure 11a) has a degradation temperature at 230–240 °C, while PPgMA (Figure 11b) already starts to degrade in the lower temperature range of 106–110 °C. The latter also shows a second degradation step starting at 360–370 °C. Evidently, an enhancement in thermal stability is achieved after modification for both PPgMASB and PPgMA (Figure 11). Moreover, a remarkable increase in the thermal stability of the modified PPgMA products is shown in Figure 11b. The thermograms of the furan-grafted product (FG0) and the cross-linked product (FG1) show a higher degradation temperature (370–380 °C) compared with PPgMA. The highest thermal stability is reached by the products annealed at 120 °C (FG1A120), indicative of a higher degree of cross-linking in the product [46].

## 4. Conclusions

This work describes the application of DA chemistry to the synthesis of cross-linked PP using furfuryl amine (FFA) and bismaleimide (BM). A two-step process is applied in the synthesis, namely the grafting of FFA in the maleated PP and further cross-linking with BM. The success of these reaction steps is confirmed by the presence of relevant peaks in the FTIR spectra, such as CH out-of-plane bending vibrations of the furan ring’s peak (γCH) at an absorption band of 730–734 cm^−1^, CH=CH of the BM aromatic ring’s stretching vibrations (υCH=CH) at an absorption band of 1510 cm^−1^, and the DA adduct (C-O-C, δDAring) at an absorption band of 1186 cm^−1^. The difference in reactivity between the two starting materials (PPgMA and PPgMASB) was evident from the changes of those FTIR peaks. It is clear that higher maleic anhydride content favors the overall reaction step, resulting in a higher degree of cross-linking in the product as quantified with the calculation of I_1510_/I_1707_ and I_1186_/I_1707_. The higher annealing temperature (T = 120 °C) favors the DA cross-linking reaction, resulting in a higher degree of cross-linking as confirmed by FTIR, the solubility test, and the rheology measurement. It is evident that partial de-cross-linking (retro DA) is observed using the rheology measurement within a temperature range of 150–200 °C, while no exothermic DA and endothermic retro DA transition can be observed in DSC results. Furthermore, the products exhibit higher melt strength, higher rigidity, and higher thermal stability compared with the starting materials. This study shows a new alternative route to synthesize cross-linked PP with highly desirable properties of the final product.

## Figures and Tables

**Table 1:** Functionalization of maleated PP with FFA, DA reaction with BM, and annealing experimental condition with the corresponding code for each product.

Reaction Steps	Polymer	Product Code	FFA Intake (mol equiv)	Temperature (°C)	BM Intake (mol equiv)	Reaction Time
A. Functionalization with	PPgMA	FG0	4	160		10 min
FFA	PPgMASB	FGSB0	4	160		10 min
B. DA cross-linking with	FG0	FG1	0.5	50	0.5	180 min
BM	FGSB0	FGSB1	0.5	50	0.5	180 min
C. Annealing step	FG1	FG1A50		50		24 h
		FG1A120		120		24 h
	FGSB1	FGSB1A50		50		24 h
		FGSB1A120		120		24 h

**Table 2:** Elemental analysis results on maleated PP and furan-grafted products.

					MA		
	N%	C%	H%	O% ^b^	mol %	wt. %	% Grafted ^a^
PPgMA	<0.01	81.93	13.70	4.37	0.091	8.9	
PPgMASB	<0.01	85.16	14.56	0.29	0.0059	0.58	
FG0	0.34	84.62	13.77	1.28			26.3
FGSB0	0.08	85.75	14.11	0.06			96.2

^a^ % grafted = mol N in furan-grafted product/mol MA in maleated PP. ^b^ The remaining composition is assumed to consist of oxygen.

**Table 3:** Intensity ratio of furan bismaleimide DA peaks.

	I_1510_/I_1707_	I_1184_/I_1707_
FG1	0.377	0.077
FG1A50	0.329	0.117
FG1A120	0.255	0.618
FGSB1	0.220	0
FGSB1A50	0.217	0
FGSB1A120	0.270	0.066

## Data Availability

Data from this study are available upon request from the corresponding author.

## Supplementary Materials

The following are available online at https://www.mdpi.com/article/10.3390/polym14061176/s1, Figure S1: The second heating and cooling cycle from DSC scan of FG1, FG1A50, and FG1A120, Figure S2: Storage (G′) modulus of PPgMASB and FGSB0 at various angular frequency (ω, rad/s) and measured at temperature of 160 °C, Figure S3: Complex viscosity (|η*|) of PPgMASB and its derivatives at various angular frequency (ω, rad/s) and measured at temperature of 160 °C.
