# Supplementary material for: Cross-Linking of Polypropylene via the Diels–Alder Reaction
Source: Polymers (Basel). 2022 Mar 15;14(6):1176. doi: 10.3390/polym14061176 (PMC8955959; doi:10.3390/polym14061176)
Supplement: Supplementary file 1 [file polymers-14-01176-s001.zip › polymers-1587615-supplementary.pdf]

Supporting Information

Article

Cross-Linking of Polypropylene via the Diels–Alder Reaction

Henky Muljana [1,2,*], Stefan Arends [2], Klaas Remerie [3], Gert Boven [3], Francesco Picchioni [2] and Ranjita K. Bose [2,*]

[1] Department of Chemical Engineering, Parahyangan Catholic University, Ciumbuleuit 94, Bandung 40141, Indonesia
[2] Department of Chemical Engineering, ENTEG, University of Groningen, Nijenborgh 4, 9747 AG Groningen, The Netherlands; stefanarends@gmail.com (S.A.); f.picchioni@rug.nl (F.P.)
[3] SABIC, Plasticslaan 1, P.O. Box 117, 4600 AC Bergen op Zoom, The Netherlands; k.remerie@home.nl (K.R.); gert.boven@sabic.com (G.B.)
* Correspondence: henky@unpar.ac.id (H.M.); r.k.bose@rug.nl (R.K.B.)

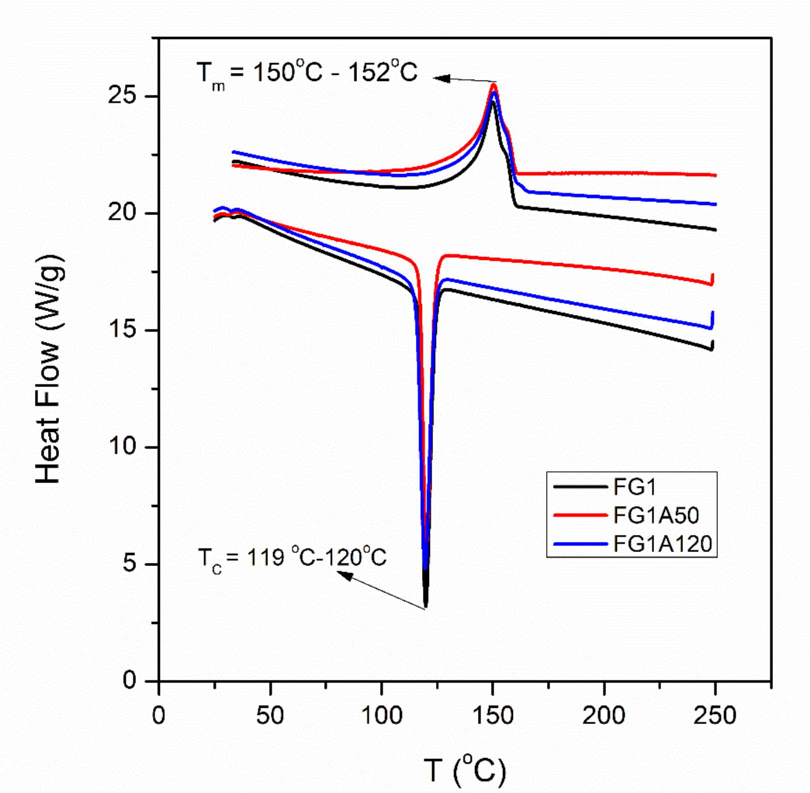

Figure S1. The second heating and cooling cycle from DSC scan of FG1, FG1A50, and FG1A120.

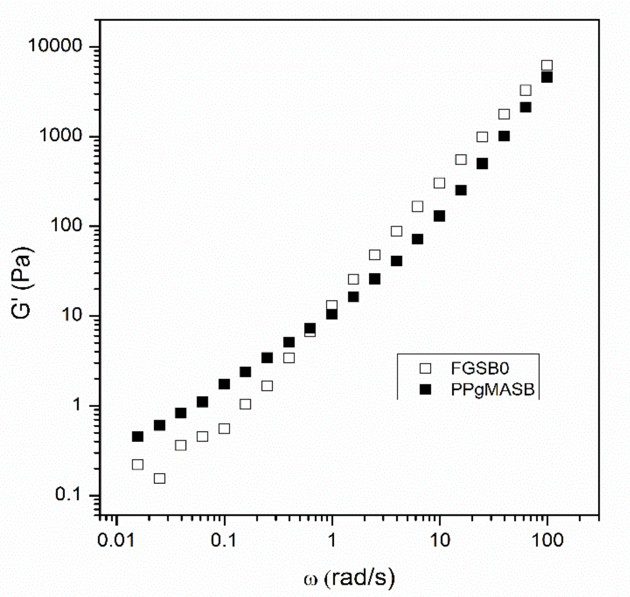

Figure S2. Storage (G′) modulus of PPgMASB and FGSB0 at various angular frequency (ω, rad/s) and measured at temperature of 160 °C.

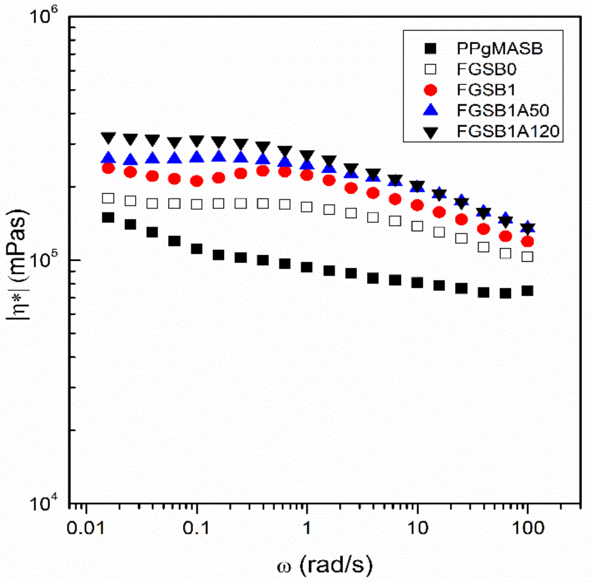

Figure S3. Complex viscosity (|η*|) of PPgMASB and its derivatives at various angular frequency (ω, rad/s) and measured at temperature of 160 °C.
